# Metabolically Induced Intestinal Inflammation: The Role of ER Stress and Autophagy in a Porcine Model of Diabetes

**DOI:** 10.21203/rs.3.rs-9655893/v1

**Published:** 2026-05-22

**Authors:** Ugljesa Malicevic, Ranko Skrbic, Devendra K. Agrawal

**Affiliations:** Western University of Health Sciences; University of Banja Luka; Western University of Health Sciences

**Keywords:** Autophagy, Cytokines, ER stress, Hyperglycemia, Intestinal inflammation, Macrophage activation, Porcine model

## Abstract

The mechanisms linking chronic hyperglycemia to intestinal inflammation and epithelial dysfunction remain incompletely understood, highlighting an important gap in our understanding of diabetes-associated gastrointestinal pathology. In this study, we investigated the effects of sustained hyperglycemia on intestinal inflammation, endoplasmic reticulum (ER) stress, and autophagy in a translational porcine model of diabetes. Diabetes was induced in Yucatan mini pigs using a high-fat, high-carbohydrate/fructose diet (HFHFD) followed by streptozotocin administration. Intestinal tissues from the terminal ileum and sigmoid colon were analyzed using histological evaluation, quantitative real-time PCR, and immunohistochemistry. Histological analysis revealed structural alterations in diabetic animals, including villous degeneration, crypt depletion, goblet-cell loss, and increased inflammatory-cell infiltration. Gene expression analysis revealed significant upregulation of inflammatory mediators (NF-κB, TNF-α, IL-6, IL-1β), inflammasome components (NLRP3), and macrophage markers (CD68, CD86, CD163). In parallel, ER stress-related genes (ORMDL3, ATF6) and autophagy-associated genes (NOD2, ULK1, ATG4a) were significantly elevated in diabetic pigs. At the protein level, increased expression of ER stress markers was confirmed in both intestinal regions, while autophagy-related proteins showed less consistent changes and did not fully reflect the observed transcriptional patterns, suggesting a potential disconnect between transcriptional activation and functional autophagic response under diabetic conditions. Chronic hyperglycemia is associated with intestinal inflammation and disruption of cellular stress pathways, including ER stress and autophagy, in a porcine model. These findings provide mechanistic insight into how chronic hyperglycemia contributes to intestinal dysfunction through coordinated alterations in inflammatory signaling, ER stress, and autophagy pathways, identifying these processes as potential targets for therapeutic intervention in diabetes-associated gastrointestinal disease.

## Introduction

Intestinal inflammation represents a key mechanism underlying gastrointestinal pathology arising from complex interactions between epithelial barrier dysfunction, immune activation, and microbial imbalance (1). Under physiological conditions, the intestinal epithelium forms a highly regulated interface between host tissues and the luminal microbiota, maintaining immune tolerance while ensuring effective defense against invading pathogens (2). Disruption of this tightly controlled balance compromises epithelial integrity, alters cytokine signaling, and promotes sustained mucosal immune activation, leading to progressive tissue damage and impaired regenerative capacity. These processes are well recognized in chronic inflammatory disorders such as inflammatory bowel disease (IBD), but increasing evidence suggests that similar mechanisms may also contribute to metabolic conditions, including diabetes, where systemic metabolic disturbances promote intestinal dysfunction (3–5). In this context, gut dysbiosis represents a key driver of intestinal inflammation, defined as a disturbance in the composition and function of the intestinal microbiota (6). Dysbiosis promotes the expansion of pathogenic microbial populations while reducing beneficial commensal species that support epithelial barrier integrity (7). As a result, microbial components, particularly lipopolysaccharide (LPS), can translocate across the compromised intestinal epithelium (8). Once in the mucosal environment, LPS activates pattern recognition receptors (PRRs), including Toll-like receptor 4 (TLR4), expressed on epithelial and immune cells, thereby initiating downstream inflammatory signaling cascades (9,10). This activation leads to the release of key pro-inflammatory cytokines, including tumor necrosis factor alpha (TNF-α), interleukin-6 (IL-6), and interleukin-1 beta (IL-1β), which are commonly elevated in conditions such as IBD and other forms of intestinal inflammation (11,12). These cytokines further amplify the inflammatory response by promoting immune cell recruitment and activation, particularly macrophages, which play a central role in sustaining mucosal inflammation. Through the production of cytokines, chemokines, and reactive oxygen species (ROS), macrophages contribute to epithelial injury and perpetuate immune dysregulation (13). Increasing evidence suggests that metabolic disturbances can also drive inflammatory alterations within the intestinal environment (14). Sustained hyperglycemia, the defining metabolic feature of diabetes mellitus (DM), has been increasingly linked to gastrointestinal dysfunction and enhanced susceptibility to mucosal inflammation (15,16). Chronic hyperglycemia contributes to oxidative stress, thereby disrupting epithelial tight junction integrity and promoting a systemic low-grade inflammatory state. These effects activate shared immune pathways implicated in both metabolic and intestinal inflammatory disease. In parallel, hyperglycemia may alter host-microbiota interactions and facilitate the translocation of bacterial products across the intestinal barrier, thereby further stimulating mucosal immune responses (17). **This bidirectional relationship between metabolic dysfunction and intestinal immune activation reflects the concept of immunometabolism, in which systemic metabolic disturbances directly influence immune signaling within mucosal tissues** (18).Obesity, a metabolic condition frequently associated with diabetes, further contributes to intestinal inflammation through chronic low-grade inflammatory signaling and alterations in gut microbiota composition (19,20). At the cellular level, metabolic disturbances such as hyperglycemia promote oxidative stress, leading to disruption of intracellular homeostasis (21). Excess reactive oxygen species and inflammatory mediators interfere with protein folding and calcium balance within the endoplasmic reticulum (ER), thereby activating the unfolded protein response (UPR) and inducing ER stress (22). Although the UPR initially acts as an adaptive mechanism aimed at restoring cellular homeostasis, persistent ER stress can exacerbate inflammatory signaling and contribute to epithelial dysfunction. In parallel, metabolic stress also affects autophagy, a key cellular process responsible for the degradation of damaged organelles and misfolded proteins. Under physiological conditions, autophagy limits inflammatory signaling and helps preserve epithelial integrity. However, dysregulation of autophagic pathways may further amplify cellular stress, enhance epithelial injury, and promote mucosal inflammation (23–25). Among the molecular regulators of ER homeostasis, ORMDL3 has gained particular attention as a regulator of UPR activation, calcium signaling, and sphingolipid metabolism, with its altered expression being linked to chronic inflammatory conditions, including IBD (26). Despite these advances, the combined effects of chronic hyperglycemia on intestinal inflammation and cellular stress pathways remain insufficiently defined. In particular, the interplay between inflammatory signaling, ER stress, and autophagy within the intestinal mucosa is not fully understood. Large animal models, particularly pigs, provide a valuable platform for studying these processes due to their close resemblance to human gastrointestinal physiology, immune responses, and metabolic regulation. In the present study, we employed a diabetic pig model to investigate how sustained hyperglycemia influences intestinal inflammation and epithelial stress pathways. We assessed the expression of key inflammatory mediators and macrophage markers, together with ER stress- and autophagy-related genes, to evaluate the impact of metabolic stress on intestinal homeostasis. This study aims to provide mechanistic insight into how chronic hyperglycemia contributes to intestinal inflammation and disrupts cellular stress pathways, with particular focus on the role of ER stress and autophagy in diabetes-associated gastrointestinal dysfunction.

## Materials and Methods

### Animal Model and Experimental Design

A total of ten female Yucatan mini pigs (n = 10), aged between 5 and 7 months and weighing 30–35 kg, were included in this study. The animals were obtained from Premier Bio-resources (Ramona, California, USA). All procedures were carried out following the Institutional Animal Care and Use Committee (IACUC) guidelines at Western University of Health Sciences (Pomona, CA), under approved protocol number #R22IACUC034. Animals were housed in the university’s Animal Resource Facility under controlled environmental conditions, including a constant room temperature of 22°C and a 12-hour light/dark cycle, as outlined in the facility’s Standard Operating Procedures. Pigs were randomly assigned to two groups: control group (n = 5) and diabetic group (n = 5). The control group was fed a normal diet (ND; 20% protein, 70% carbohydrates, 10% fat), while the diabetic group received a high-fat, high-carbohydrate/fructose diet (HFHFD; 45% carbohydrates-fructose, 5% protein, 45% fat). Both groups had *ad libitum* access to water. After two months on the HFHFD, diabetes was induced in the experimental group by intravenous (i.v.) injection of streptozotocin (STZ) at a dose of 50 mg/kg, dissolved in 0.1 M citrate buffer (pH 4.4). A second STZ injection (50 mg/kg i.v.) was administered seven days later. Control animals received an equivalent volume of vehicle (0.50 mL/kg of citrate buffer i.v.) at the same time points. Blood glucose levels were monitored regularly using a validated veterinary glucometer, and animals with sustained fasting blood glucose levels exceeding 120 mg/dL were classified as diabetic. Upon confirmation of diabetes, animals remained on their respective diets for an additional two months. At the study endpoint, pigs were euthanized under surgical anesthesia via intravenous administration of a single dose of Euthasol solution, containing sodium pentobarbital and sodium phenytoin. Euthanasia was confirmed by the absence of cardiac and respiratory activity for a minimum of 10 minutes before tissue collection. Following confirmed euthanasia, tissues from the small intestine (terminal ileum) and large intestine (sigmoid colon) were collected. A portion of each sample was fixed in 10% neutral buffered formalin for histopathological analysis, while the remaining tissue was stored in pre-labeled tubes containing RNAlater solution to preserve RNA integrity for downstream total RNA isolation.

### Tissue Processing, Staining, and Histopathological Analysis

Following fixation in 10% neutral buffered formalin for 48 hours, intestinal tissue samples were processed using a Tissue-Tek VII automated tissue processor (Sakura Finetek, Torrance, CA, USA). Samples were dehydrated in ethanol, cleared with xylene, and embedded in paraffin wax. The tissues were then sectioned at a thickness of 7 μm using a Leica RM2265 rotary microtome (Leica^™^, Wetzlar, Germany), mounted on glass slides, and incubated at 60°C for one hour to ensure optimal adhesion. Before staining, paraffin sections were deparaffinized in xylene, rehydrated through a descending ethanol gradient, and stained with hematoxylin and eosin (H&E) following established laboratory procedures. Stained slides were scanned and visualized using a Leica DM6 light microscope (Leica^™^, Wetzlar, Germany). Tissue sections were examined at 5× and 20× magnifications to assess histopathological alterations. For each section, three to five high-magnification images were captured for documentation and analysis.

### RNA Extraction, cDNA Synthesis, and Quantitative Real-Time PCR

Approximately 50 mg of intestinal tissue from each sample was used for total RNA extraction using TRIzol reagent (T9424, Millipore Sigma, Burlington, MA, USA), following the manufacturer’s instructions. The resulting RNA pellets were resuspended in 30 μL of nuclease-free water (BP561–1, Thermo Fisher Scientific, Waltham, MA, USA). RNA concentration and purity were determined using a NanoDrop 2000 spectrophotometer (Thermo Fisher Scientific, Waltham, MA, USA). RNA purity was assessed by the A260/280 ratio. For complementary DNA (cDNA) synthesis, 2 μg of total RNA per sample was reverse-transcribed using the AzuraQuant^™^ cDNA Synthesis Kit (AZ-1996, Azura Genomics Inc., Raynham, MA, USA), following the manufacturer’s protocol and utilizing a T100^™^ Thermal Cycler (Bio-Rad Laboratories, Hercules, CA, USA). Quantitative real-time PCR (qPCR) was performed in triplicate for each sample using the AzuraView^™^ GreenFast qPCR Blue Mix LR (AZ-2350, Azura Genomics Inc., Raynham, MA, USA) in a final reaction volume of 10 μL. Amplification was carried out on a C1000^™^ Thermal Cycler (Bio-Rad Laboratories, Hercules, CA, USA) under the following cycling conditions: initial denaturation at 95°C for 3 minutes, followed by 39 cycles of 95°C for 10 seconds and 60°C for 30 seconds, concluding with a melting curve analysis to verify amplification specificity. Primers for target genes and the housekeeping gene 18S rRNA (used for normalization) were obtained from Integrated DNA Technologies (Coralville, IA, USA), with sequences listed in Table 1. Relative gene expression levels were calculated using the 2^−ΔΔ^C_T_ method.

### Immunohistochemistry (IHC)

Immunohistochemical analysis was performed to evaluate protein expression in formalin-fixed, paraffin-embedded intestinal tissue sections. Samples were first deparaffinized in xylene and rehydrated through a graded ethanol series. Antigen retrieval was carried out by heating the sections in 1% citrate buffer (C9999–1000ML, Millipore Sigma, Burlington, MA, USA) using a commercial steamer for 20–30 minutes, followed by cooling at room temperature for 30–45 minutes. Slides were then rinsed in 1× phosphate-buffered saline (PBS; BP39920, Thermo Fisher Scientific, Waltham, MA, USA) for 5 minutes. To block endogenous peroxidase activity, sections were incubated in 3% hydrogen peroxide (H1009, Millipore Sigma) for 15 minutes at room temperature, followed by two PBS washes. Non-specific binding was blocked by incubating the sections with species-specific normal serum for 1 hour at room temperature: Normal Goat Serum (S-1000–20) for rabbit primary antibodies and Normal Horse Serum (S-2000–20) for mouse primary antibodies (Vector Laboratories). After blocking, sections were incubated overnight at 4°C with primary antibodies diluted in antibody diluent buffer, using concentrations optimized through prior titration. The following day, slides were washed twice in PBS and incubated with species-appropriate secondary antibodies for 1 hour at room temperature. After two additional PBS washes, signal amplification was performed using the VECTASTAIN^®^ ABC-HRP Kit (PK-4000, Vector Laboratories, Newark, CA, USA) for 30 minutes. Chromogenic development was conducted using the AEC Substrate Kit (3-amino-9-ethylcarbazole; SK-4200, Vector Laboratories) for 3–5 minutes or until optimal staining intensity was achieved. The reaction was stopped by rinsing in tap water. Slides were counterstained with hematoxylin (2 dips), rinsed in water, and mounted using ADVANTAGE Mounting Media (NB300A, Innovex Biosciences, Pinole, CA, USA). Positive and negative controls were included for each antibody to validate staining specificity. Stained sections were scanned using a Leica DM6 light microscope (Leica^™^, Wetzlar, Germany) with a calibrated scale bar of 100 μm. For semi-quantitative analysis, at least three randomly selected high-power fields per tissue section were captured and analyzed using Fiji ImageJ software (version 1.54J, NIH, USA) to determine the mean staining intensity and percentage of positively stained area.

## Results

Induction of hyperglycemia resulted in a clear increase in fasting glucose levels compared to baseline measurements obtained before the initiation of the experimental protocol and dietary regimen. Fasting glucose values measured after diabetes induction exhibited substantial inter-individual variability. Insulin levels remained relatively moderate, while homeostatic model assessment of insulin resistance (HOMA-IR) values indicated varying degrees of insulin resistance (IR) among animals, reflecting a heterogeneous metabolic response.

### Histological analysis

**Figure 2:**
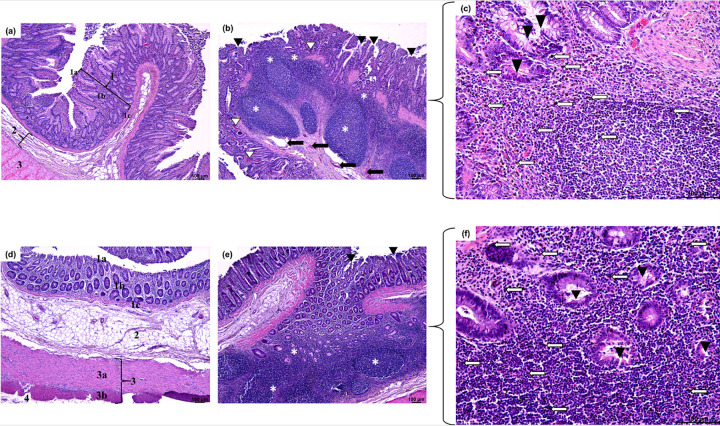
Histological alterations in small and large intestinal tissues of control and diabetic pigs, stained with hematoxylin and eosin (H&E). Panels (a, b, d, and e): Cross-sections of the small and large intestine at 5× magnification. Panels (c, f): Cross-sections of the small and large intestine at 20× magnification. Scale bars: 100 μm (all panels). Panel (a) – Cross-section of the small intestine from a control pig. The mucosa (1) is lined by simple columnar epithelium with a well-defined brush border, composed of absorptive enterocytes and mucus-secreting goblet cells (1a). Intestinal glands (crypts of Lieberkühn) extend into the mucosa and display normal architecture within the lamina propria (1b), supported by the muscularis mucosae (1c). The submucosa (2) consists of dense connective tissue rich in blood vessels and lymphatics. The muscularis externa (3) with its inner circular smooth-muscle layer is present. Panel (b) – Cross-section of the small intestine from a diabetic pig. The mucosa shows villous-tip degeneration and depletion of intestinal glands (black arrowhead). Mild hemorrhage is present multifocally within the lamina propria (white arrowhead). Inflammatory infiltrates surround and disrupt the muscularis mucosae, forming prominent lymphoid aggregates in the submucosa (*). Mild edema and congestion are also evident in the submucosa (black arrow). Panel (d) – Cross-section of the large intestine from a control pig. The mucosa (1) is characterized by a smooth surface lined by simple columnar epithelium composed of absorptive cells and abundant mucus-secreting goblet cells (1a). Numerous closely packed, straight tubular glands (crypts of Lieberkühn) extend into the lamina propria (1b). The lamina propria is highly vascularized and supported by the muscularis mucosae (1c). The submucosa (2) consists of dense connective tissue rich in blood vessels. The muscularis externa (3) is composed of inner circular (3a) and outer longitudinal (3b) smooth-muscle layers. The serosa (4) is formed by loose connective tissue lined with mesothelium. Panel (e) – Cross-section of the large intestine from a diabetic pig. The mucosal epithelium is largely preserved, with only mild multifocal disintegration (black arrowhead). The lamina propria shows increased cellularity, including immune and inflammatory cells that extend toward the muscularis mucosae and form large basophilic lymphoid aggregates in the submucosa (*). The submucosa, muscularis externa, and serosa remain intact. Panels (c, f) – Higher-magnification photomicrographs demonstrating mild crypt depletion (black arrowheads) with goblet-cell loss and increased lamina propria cellularity due to lymphocytes, neutrophils, and lipofuscin-like pigment-filled macrophages (white arrows).

**Figure 3:**
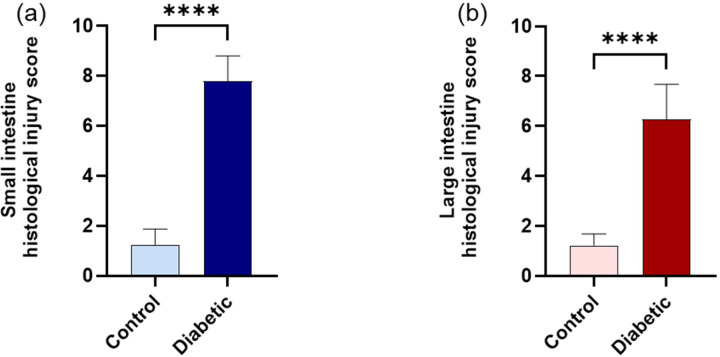
Quantitative assessment of histological injury in the small and large intestines of control and diabetic pigs. **Panel (a)**represents the small intestine, and panel **(b)**represents the large intestine. Data are presented as mean ± SD. Statistical significance was determined using Student’s *t*-test.

Histological evaluation of intestinal tissues revealed clear differences between the control and diabetic pigs. In control animals, both the small and large intestines exhibited well-preserved architecture, characterized by intact villi in the small intestine, well-organized intestinal glands (crypts of Lieberkühn), and normal epithelial lining containing absorptive columnar cells and mucus-secreting goblet cells. The lamina propria, submucosa, muscularis externa, and serosa displayed normal structural organization without evident pathological alterations (Fig. 2a, d). In contrast, intestinal tissues from diabetic pigs showed marked structural and cellular alterations in both intestinal regions. These changes included mucosal degeneration, reduced glandular integrity, and increased infiltration of inflammatory cells within the intestinal wall (Fig. 2b, e). The deeper layers, including the muscularis externa and serosa, remained largely preserved. At higher magnification, these alterations were further characterized by mild crypt depletion, goblet-cell loss, and increased cellularity of the lamina propria (Fig. 2c, f). These findings were further supported by quantitative histological injury scoring, which demonstrated increased injury in diabetic animals compared with controls (Fig. 3).

**Panel (a)** – Cross-section of the small intestine from a control pig. The mucosa (1) is lined by simple columnar epithelium with a well-defined brush border, composed of absorptive enterocytes and mucus-secreting goblet cells (1a). Intestinal glands (crypts of Lieberkühn) extend into the mucosa and display normal architecture within the lamina propria (1b), supported by the muscularis mucosae (1c). The submucosa (2) consists of dense connective tissue rich in blood vessels and lymphatics. The muscularis externa (3) with its inner circular smooth-muscle layer is present.

**Panel (b)** – Cross-section of the small intestine from a diabetic pig. The mucosa shows villous-tip degeneration and depletion of intestinal glands (black arrowhead). Mild hemorrhage is present multifocally within the lamina propria (white arrowhead). Inflammatory infiltrates surround and disrupt the muscularis mucosae, forming prominent lymphoid aggregates in the submucosa (*). Mild edema and congestion are also evident in the submucosa (black arrow).

**Panel (d)** – Cross-section of the large intestine from a control pig. The mucosa (1) is characterized by a smooth surface lined by simple columnar epithelium composed of absorptive cells and abundant mucus-secreting goblet cells (1a). Numerous closely packed, straight tubular glands (crypts of Lieberkühn) extend into the lamina propria (1b). The lamina propria is highly vascularized and supported by the muscularis mucosae (1c). The submucosa (2) consists of dense connective tissue rich in blood vessels. The muscularis externa (3) is composed of inner circular (3a) and outer longitudinal (3b) smooth-muscle layers. The serosa (4) is formed by loose connective tissue lined with mesothelium.

**Panel (e)** – Cross-section of the large intestine from a diabetic pig. The mucosal epithelium is largely preserved, with only mild multifocal disintegration (black arrowhead). The lamina propria shows increased cellularity, including immune and inflammatory cells that extend toward the muscularis mucosae and form large basophilic lymphoid aggregates in the submucosa (*). The submucosa, muscularis externa, and serosa remain intact.

**Panels (c, f)** – Higher-magnification photomicrographs demonstrating mild crypt depletion (black arrowheads) with goblet-cell loss and increased lamina propria cellularity due to lymphocytes, neutrophils, and lipofuscin-like pigment-filled macrophages (white arrows).

### Gene Expression Analysis

**Figure 4:**
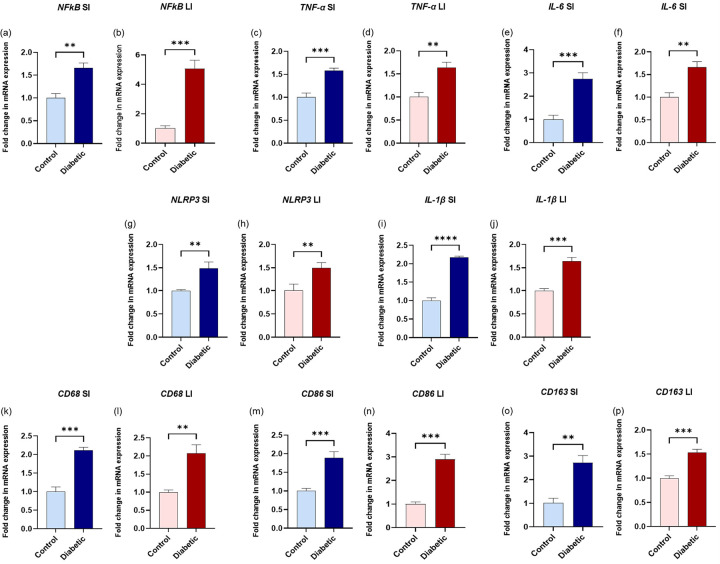
Fold change of genes involved in inflammatory signaling and macrophage activation across the intestines of control and diabetic pigs. Blue bars represent the small intestine (SI), while red bars represent the large intestine (LI). *NF-κB* (a, b), *TNF-α* (c, d), *IL-6* (e, f), *NLRP3* (g, h), *IL-1β* (i, j), *CD68* (k, l), *CD86* (m, n), and *CD163* (o, p), with each pair representing the small and large intestine, respectively. Gene expression was quantified by qPCR and normalized to a housekeeping gene. Statistical comparisons between the control and diabetic groups were performed using Student’s *t*-test. Data are presented as mean ± SD. Statistical significance: *p* < 0.05, *p* < 0.01, *p* < 0.001, *p* < 0.0001.

To investigate the relationship between chronic inflammation and cellular stress responses in diabetic conditions, we analyzed the expression of key pro-inflammatory cytokines and macrophage markers in the intestines of this hyperglycemic pig model. Because inflammatory signaling is closely linked to intracellular stress pathways, including ER stress and autophagy, we also assessed the expression of ER stress-related genes and autophagy markers to evaluate potential disruptions in cellular homeostasis. Expression analysis revealed significant upregulation of several inflammatory mediators in the intestines of diabetic pigs. *NF-κB*, a central transcription factor regulating inflammatory gene expression, was significantly increased in the small intestine (p < 0.01) and showed an even greater elevation in the large intestine (p < 0.001) (Fig. 4a, b). Similarly, *TNF-α* and *IL-6* were markedly elevated in the small intestine (p < 0.001) and remained significantly increased in the large intestine (p < 0.01) (Fig. 4c–f). The inflammasome component *NLRP3* was consistently upregulated in both intestinal segments (p < 0.01) (Fig. 4g, h). Among the cytokines analyzed, *IL-1β* exhibited the most pronounced increase, particularly in the small intestine (p < 0.0001) (Fig. 4i, j). To further characterize immune cell involvement, transcripts associated with macrophage populations were evaluated. The pan-macrophage marker *CD68* was significantly increased in diabetic pigs, indicating enhanced mononuclear phagocyte presence in the intestinal mucosa (p < 0.001 in the small intestine, p < 0.01 in the large intestine) (Fig. 4k, l). The M1 polarization marker *CD86* was strongly upregulated in both intestinal segments (p < 0.001) (Fig. 4m, n), suggesting a predominance of pro-inflammatory macrophage activity. In addition, the M2 marker *CD163* showed moderate upregulation in the small intestine (p < 0.01) and a more pronounced increase in the large intestine (p < 0.001) (Fig. 4o, p), suggesting that anti-inflammatory and tissue repair mechanisms may be concurrently activated, possibly as a compensatory response to ongoing inflammation.

**Figure 5:**
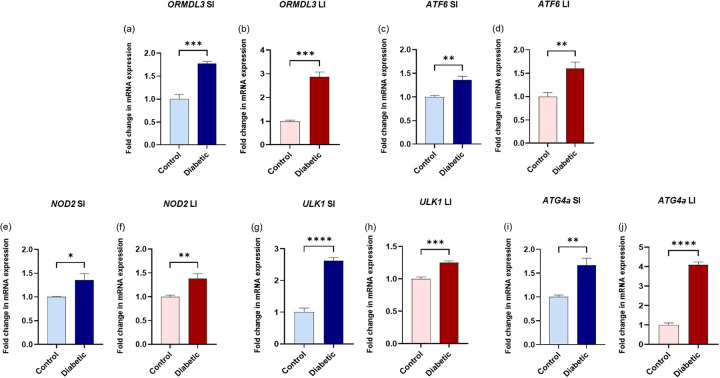
Fold change of ER stress- and autophagy-related genes across the intestines of control and diabetic pigs. Blue bars represent the small intestine (SI), while red bars represent the large intestine (LI). *ORMDL3*(a, b), *ATF6* (c, d), *NOD2* (e, f), *ULK1* (g, h), and *ATG4a*(i, j), with each pair representing the small and large intestine, respectively. Gene expression was quantified by qPCR and normalized to a housekeeping gene. Statistical comparisons between control and diabetic groups were performed using Student’s *t*-test. Data are presented as mean ± SD. Statistical significance: *p* < 0.05, *p* < 0.01, *p* < 0.001, *p* < 0.0001.

To assess cellular stress responses, we next examined the expression of ER stress-related genes. *ORMDL3*, a regulator of ER homeostasis and sphingolipid metabolism, was significantly upregulated in both the small intestine (p < 0.001) and the large intestine (p < 0.001) (Fig. 5a, b). Similarly, *ATF6* as one of the key sensors of the unfolded protein response, showed increased expression in both intestinal segments (p < 0.01 in the small intestine, p < 0.01 in the large intestine) (Fig. 5c, d). Autophagy-related genes were also affected by hyperglycemic conditions. *NOD2*, which links microbial sensing to autophagy activation, showed moderate upregulation in the small intestine (p < 0.05) and a more pronounced increase in the large intestine (p < 0.01) (Fig. 5e, f). *ULK1* as a central regulator of autophagosome formation, was markedly elevated in diabetic pigs (p < 0.0001 in the small intestine, p < 0.001 in the large intestine) (Fig. 5g, h). In addition, *ATG4a* which is involved in autophagosome maturation, was significantly increased in both intestinal segments (p < 0.01 in the small intestine, p < 0.0001 in the large intestine) (Fig. 5i, j).

In line with these observations, protein expression analysis showed increased ER stress-related markers in the intestines of diabetic pigs, while autophagy-related proteins exhibited less pronounced changes compared to gene expression results.

### Immunohistochemical (IHC) analysis

**Figure 6:**
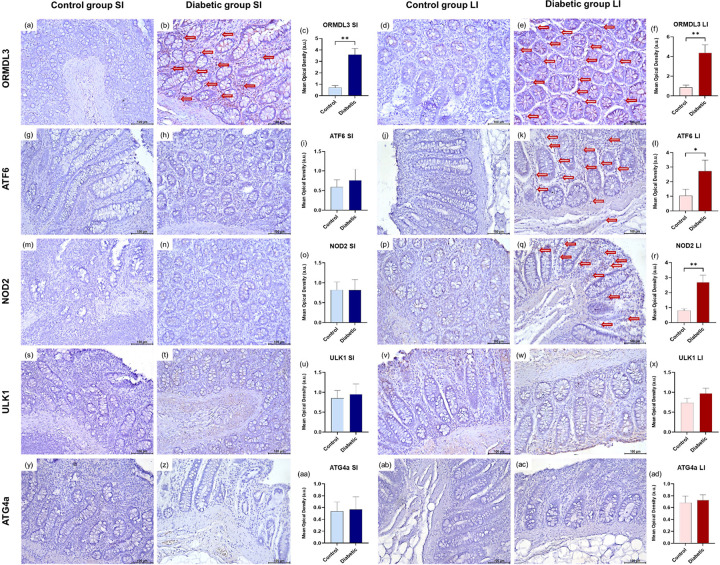
Immunohistochemical (IHC) staining and quantitative analysis of ORMDL3, ATF6, NOD2, ULK1, and ATG4a protein expression in the small and large intestines of control and diabetic pigs. For each protein, representative IHC images of control and diabetic samples are shown for the small intestine, followed by the corresponding quantitative analysis of mean optical density. The same sequence is presented for the large intestine. ORMDL3 expression is shown in panels (a–f), ATF6 in (g–l), NOD2 in (m–r), ULK1 in (s–x), and ATG4a in (y–ad). In each set of panels, the first two images represent control and diabetic samples, respectively, followed by the corresponding quantification. Blue bars represent data from the small intestine (SI), and red bars represent data from the large intestine (LI). Data are presented as mean ± SD. Statistical comparisons between control and diabetic groups were performed using Student’s *t-test*. Statistical significance: *p < 0.05, **p < 0.01, ***p < 0.001, ****p < 0.0001.

To further validate the transcriptional findings, protein expression of ER stress and autophagy-related markers was assessed in the small and large intestines of control and diabetic pigs using immunohistochemistry. Analysis of ORMDL3 expression revealed increased protein levels in diabetic animals in both intestinal regions. In the small intestine, ORMDL3 expression was significantly elevated in diabetic pigs compared to controls (p < 0.01) (Fig. 6a–c), and a similar increase was observed in the large intestine (p < 0.01) (Fig. 6d–f). Evaluation of ATF6 expression demonstrated no significant difference between control and diabetic groups in the small intestine (Fig. 6g–i). In contrast, ATF6 expression was modestly but significantly increased in the large intestine of diabetic pigs compared to controls (p < 0.05) (Fig. 6j–l). To further characterize autophagy-related responses, protein expression of NOD2, ULK1, and ATG4a was analyzed. NOD2 expression did not differ between control and diabetic groups in the small intestine (Fig. 6m–o); however, a significant increase was observed in the large intestine of diabetic pigs compared to controls (p < 0.01) (Fig. 6p–r). In contrast, no significant differences were detected in ULK1 expression between control and diabetic groups in either the small (Fig. 6s–u) or large intestine (Fig. 6v–x). Similarly, ATG4a expression remained unchanged across both intestinal regions, with no significant differences between experimental groups (Fig. 6y–ad). Our findings indicate that while ER stress–related proteins were upregulated under diabetic conditions, autophagy-related protein expression did not fully parallel the observed transcriptional changes.

## Discussion

**Figure 7:**
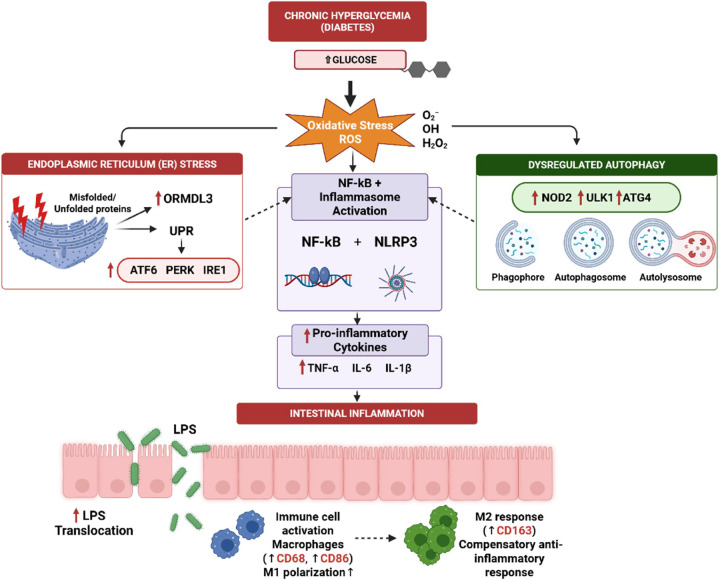
Schematic representation of the proposed pathogenic mechanisms linking chronic hyperglycemia to intestinal inflammation in the diabetic intestine. Sustained hyperglycemia is associated with oxidative stress, which may activate NF-κB and NLRP3 inflammasome signaling, leading to pro-inflammatory cytokine production and mucosal immune activation. In parallel, hyperglycemia is linked to endoplasmic reticulum stress through ORMDL3 upregulation and UPR activation, while simultaneously dysregulating autophagy-related pathways, including NOD2, ULK1, and ATG4. Together, these interconnected mechanisms may contribute to epithelial barrier dysfunction, macrophage polarization, and sustained intestinal inflammation.

The present study demonstrates that chronic hyperglycemia is associated with significant and coordinated alterations in intestinal structure, inflammatory signaling, and cellular stress pathways in a porcine model, affecting both the terminal ileum and sigmoid colon. These regions were selected due to their distinct physiological and immunological characteristics, as they represent key segments of the small and large intestine, enabling assessment of region-specific responses to hyperglycemia. The terminal ileum plays a key role in mucosal immune responses and antigen exposure, while the sigmoid colon is characterized by a dense and metabolically active microbiota (2, 27, 28). Importantly, the consistency of findings across both intestinal segments, despite their distinct physiological roles, suggests that hyperglycemia-associated intestinal dysfunction is not strictly region-specific and may reflect a systemic disruption of intestinal homeostasis under chronic metabolic stress. The induction of hyperglycemia in the present study resulted in a clear elevation of fasting glucose levels, confirming the successful establishment of the experimental diabetic model. Despite this, insulin concentrations remained within a moderate range, while HOMA-IR values indicated variable degrees of insulin resistance (IR) among animals. This pattern suggests that the metabolic phenotype induced in this model reflects a combination of partial β-cell dysfunction and IR, which is characteristic of low-dose STZ models combined with dietary manipulation (29). The relatively high inter-individual variability observed, particularly in glucose levels, is consistent with previous studies demonstrating heterogeneous responses to STZ-induced diabetes (30, 31). Such variability may reflect differences in individual susceptibility to β-cell damage, metabolic adaptation, and the balance between IR and residual insulin secretion (32). Importantly, this heterogeneity may have influenced downstream intestinal and molecular responses, providing a relevant framework for interpreting the observed alterations in intestinal structure, inflammation, and gene expression. These metabolic alterations were accompanied by marked structural and inflammatory changes in the intestines of diabetic pigs, including villous degeneration, crypt depletion, goblet-cell loss, and increased inflammatory-cell infiltration, while the deeper layers remained largely preserved. These findings are consistent with previous reports indicating that metabolic disturbances, particularly hyperglycemia, may impair epithelial integrity and promote mucosal inflammation (15, 33, 34). Loss of goblet cells and disruption of epithelial architecture are particularly relevant, as they impair mucus barrier function and further compromise epithelial integrity, increasing susceptibility to luminal antigens and microbial products (35)(36). Increased lamina propria cellularity and lymphoid aggregate formation were observed, indicating enhanced immune activation, a hallmark of chronic mucosal inflammation. In line with these structural alterations, gene expression analysis revealed a pronounced upregulation of inflammatory mediators in the intestines of diabetic pigs, characterized by increased expression of NF-κB, TNF-α, IL-6, IL-1β, and NLRP3. Activation of NF-κB signaling plays a central role in coordinating intestinal inflammation by regulating the expression of cytokines and inflammasome-related components (37). Elevated levels of TNF-α, IL-6, and IL-1β further reflect an amplified inflammatory response, as these cytokines are key mediators of mucosal immune activation and epithelial barrier disruption (38, 39). Consistent with this inflammatory profile, increased expression of CD68 and CD86 indicates enhanced macrophage infiltration and polarization toward a pro-inflammatory M1 phenotype, contributing to sustained cytokine production and tissue injury. Interestingly, the concurrent upregulation of CD163 suggests the presence of M2 macrophages, indicating that anti-inflammatory and tissue repair mechanisms may be activated alongside ongoing inflammation, potentially reflecting a compensatory response. This dual macrophage response highlights the complexity of immune regulation in the diabetic intestine and supports the concept of a dynamically regulated inflammatory microenvironment under metabolic stress conditions (18)(Fig. 7).

Alongside these inflammatory alterations, our findings indicate a clear activation of endoplasmic reticulum (ER) stress in the intestinal mucosa of diabetic pigs. The significant upregulation of ORMDL3 and ATF6 at the transcriptional level indicates activation of the unfolded protein response (UPR), a key adaptive mechanism triggered under conditions of cellular stress (22). ORMDL3 has been implicated in the regulation of ER homeostasis and inflammatory signaling, particularly in epithelial and immune cells, where its overexpression has been associated with increased susceptibility to inflammatory disorders, including IBD (26). Similarly, ATF6 functions as a central sensor of ER stress and plays a critical role in restoring protein-folding capacity under stress conditions (22, 40). Consistent with these mechanisms, our data show increased ORMDL3 expression at both the gene and protein levels in the small and large intestine, supporting its role in linking metabolic stress to inflammatory signaling and epithelial dysfunction (Fig. 7). In contrast, ATF6 protein expression was predominantly increased in the large intestine, despite transcriptional upregulation in both segments, suggesting region-specific activation of ER stress pathways. This difference may be related to the higher microbial load in the colon, which can further amplify ER stress through microbial-derived signals and immune activation. These findings are partially consistent with our previous observations in a rat model, where ATF6 expression was increased at both the gene and protein levels in the small intestine under hyperglycemic conditions (41). However, the present study reveals a more region-specific pattern at the protein level, with predominant upregulation of ATF6 in the large intestine, suggesting that species-specific or tissue-specific regulatory mechanisms may differentially shape ER stress responses along the intestinal tract. Autophagy-related pathways were also transcriptionally activated in the intestines of diabetic pigs, as evidenced by the upregulation of NOD2, ULK1, and ATG4a. These molecules play key roles in microbial sensing, autophagosome formation, and autophagic processing, and are essential for maintaining intestinal homeostasis (42–44). In particular, NOD2 is closely involved in host-microbiota interactions and intestinal immune regulation, with its dysregulation being strongly linked to Crohn's disease (45, 46). However, despite clear transcriptional activation, these changes were not consistently reflected at the protein level. In the present study, ULK1 and ATG4a expression remained unchanged in both intestinal segments, while NOD2 protein expression was increased predominantly in the large intestine. This discrepancy between gene and protein expression suggests that autophagy is not uniformly activated, but rather dysregulated under hyperglycemic conditions, highlighting a potential disconnect between transcriptional activation and functional autophagic response in the diabetic intestine. Such findings may indicate impairment at the post-transcriptional or post-translational level, potentially involving altered protein turnover, translational control, or defective autophagic flux. Similar patterns have been described in chronic metabolic stress, where transcriptional activation of autophagy-related genes does not necessarily translate into effective autophagic function, ultimately contributing to cellular dysfunction and sustained inflammation (47, 48). Overall, this pattern suggests that although intestinal cells initiate autophagic responses under metabolic stress, these mechanisms remain functionally insufficient, highlighting autophagy dysregulation as a contributing factor to intestinal pathology under diabetic conditions (Fig. 7). Beyond their individual contributions to intestinal pathology, the concurrent activation of ER stress and autophagy-related pathways observed in this study suggests that these processes likely represent interconnected cellular responses to hyperglycemia-associated metabolic stress rather than independent phenomena. The strong positive correlations between ORMDL3, ATF6, and autophagy-related genes further support the existence of a coordinated ER stress-autophagy axis in the diabetic intestine (41, 49). This functional relationship is particularly relevant in the context of intestinal homeostasis, where effective resolution of ER stress through autophagic clearance of misfolded proteins is essential for preserving epithelial integrity and mucosal immune balance (25). Taken together, our findings highlight a complex relationship between metabolic stress, inflammation, and cellular stress pathways in the diabetic intestine. Chronic hyperglycemia is closely linked to epithelial damage, immune activation, ER stress, and dysregulated autophagy, ultimately leading to intestinal dysfunction. The use of a porcine model adds significant translational value, as its gastrointestinal physiology, immune responses, and metabolic regulation closely resemble those observed in humans, allowing for a more clinically relevant interpretation of intestinal alterations under diabetic conditions. In particular, ORMDL3 and associated ER stress pathways emerge as potentially important contributors to this dysfunction, representing a molecular axis that warrants further investigation as a therapeutic target. The interplay of inflammatory signaling, ER stress, and autophagy dysregulation observed in this model suggests that effective management of diabetes-associated intestinal disease may require therapeutic strategies that address these interconnected mechanisms rather than targeting inflammation alone.

## Conclusion

Chronic hyperglycemia is associated with structural and molecular changes in the intestinal mucosa, including epithelial damage, increased inflammatory signaling, activation of ER stress, and altered autophagy. These changes were observed in both intestinal segments, with differences at the protein level, particularly in ER stress and autophagy-related markers. Taken together, the histological, gene expression, and protein data show that metabolic stress not only contributes to inflammation but also disrupts mechanisms that maintain cellular homeostasis. The observed discrepancy between gene and protein expression in autophagy-related pathways suggests that increased transcription does not necessarily translate into effective functional responses, pointing to impaired cellular adaptation under diabetic conditions. Given the close physiological and immunological similarities between porcine and human gastrointestinal systems, these findings provide a translationally relevant framework for understanding the contribution of chronic hyperglycemia to intestinal dysfunction. Collectively, the results suggest that effective management of diabetes-associated intestinal disease may require therapeutic strategies that simultaneously target inflammatory signaling, ER stress, and autophagic dysregulation, rather than addressing these pathways in isolation.

### Study limitations

Although the porcine model closely resembles human gastrointestinal physiology, it does not fully capture the complexity of human diabetes, which may limit direct clinical translation. The analysis was restricted to a single time point of chronic hyperglycemia, preventing assessment of the temporal progression of intestinal alterations and the dynamic interplay between inflammation, ER stress, and autophagy. In addition, while changes in autophagy-related gene and protein expression were observed, functional autophagic flux was not directly assessed, making it difficult to determine whether these alterations reflect effective or impaired autophagy. Furthermore, the study focused on the terminal ileum and sigmoid colon, which, although selected as representative and functionally important regions of the small and large intestine, may not fully reflect region-specific responses along the entire intestinal tract, highlighting the need for future studies to include additional segments.

## Figures and Tables

**Figure 1:**
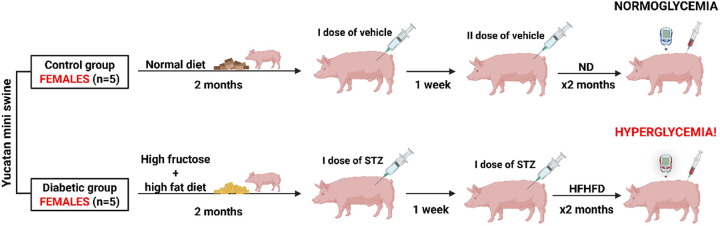
Protocol for diabetes induction and dietary regimen of experimental animals during the study. Yucatan mini pigs: STZ - streptozotocin (50 mg/kg i.v.); vehicle - citrate buffer (0.50 mL/kg i.v.); Normal diet (ND); High fat, high carbohydrate/fructose diet (HFHFD); i.v. - intravenous.

**Table 1:** The forward and reverse oligonucleotide sequences used for RT-qPCR amplification of target genes. The 18S ribosomal RNA gene (*18S*) served as the housekeeping control for normalization. The analyzed genes: nuclear factor kappa B (*NFκB*), tumor necrosis factor alpha (*TNF-α*), interleukin 6 (*IL-6*), NLR family pyrin domain containing 3 (*NLRP3*), interleukin 1 beta *(IL-1β*), cluster of differentiation 68 (*CD68*), *CD86*, *CD163*, orosomucoid-like protein 3 (*ORMDL3*), activating transcription factor 6 (*ATF6*), nucleotide-binding oligomerization domain-containing protein 2 (*NOD2*), unc-51-like autophagy activating kinase 1 (*ULK1*), and autophagy-related 4 cysteine peptidase A (*ATG4a*).

Gene name	Forward primer	Reverse primer
*NFkB*	5’- GCT ACC CTG GCA CAG AAA TTA - 3’	5’- CCT CCA CCA GCT CCT TTA TTG - 3’
*TNF-α*	5’- TTC CTC ACT CAC ACC ATC AGC C –3’	5’- GGT AGA TGG GTT CGT ACC AGG A –3’
*IL-6*	5’- GGA GAC CTG CTT GAT GAG AAT C –3’	5’- CAG CCT CGA CAT TTC CCT TAT - 3’
*NLRP3*	5’- CGA GAC GTG ACA GTT CTT CTT - 3’	5’- GGA CGT TCT CTC CTG GTT TAC - 3’
*IL-1β*	5’- TGC ATG AGC TTT GTG CAA GGA G –3’	5’- AGG GTG GGC GTG TTA TCT TTC A –3’
*CD68*	5’- TCT TGT CCC AGT GAC CAA AC –3’	5’- CGG ATG ATG CAG AAG GTA ACA - 3’
*CD86*	5’- GTA TCA ATC AGG GTG TCT CTT CC –3’	5’- ACA GGT GGC TTT GCA TCT AT –3’
*CD163*	5’- CCG TCT GTG ATT CTG ACT TCT C –3’	5’- CTG TCC ACT TCC TTC TCC AAA - 3’
*ORMDL3*	5’- ACT GGG AGC AGA TGG ACT AT –3’	5’- CTG GTC GTA CTT GGT GTA GAA G –3’
*ATF6*	5’- ACT GGA GAG TAG GTG AGA GAA G –3’	5’- TTC CAA GTA GAT GGG TGG ATT G –3’
*NOD2*	5’- CTC ACC TTA AGG AGG GTT GTT - 3’	5’- GAG TGG AGG AAC CAG ATG TTA G –3’
*ULK1*	5’- CGT GTT TCT CTC TTG CGA TAC T –3’	5’- CTT AAG TCA CGG GTG ATG AGA C –3’
*ATG4a*	5’- GTT GAC GAC CAG ACT TTC CA –3’	5’- CAA GGC TAC ACC AGC TAT CAA - 3’
*18s*	5’- CCC ACG GAA TCG AGA AAG AG –3’	5’- TTG ACG GAA GGG CAC CA –3’

**Table 2:** The table provides detailed information on the primary and secondary antibodies used for immunohistochemistry (IHC), including their catalog numbers and dilution ratios. The primary antibodies target ORMDL3, ATF6, ULK1, NOD2, and ATG4a with corresponding secondary antibodies (anti-rabbit or anti-mouse) selected based on the host species of each primary antibody.

Antibody	Catalog number	IHC dilution
**Primary antibody**		
**ORMDL3**	LS-B9583	**1:100**
**ATF6**	NBP1-40256	**1:100**
**NOD2**	NB100-524	**1:100**
**ULK1**	MA5-32699	**1:100**
**ATG4a**	MA5-37669	**1:50**
**Secondary antibody**		
**Anti-rabbit**	BP-9100-50	**Ready-to-use**
**Anti-mouse**	BP-2000-50	**Ready-to-use**

**Table 3:** Metabolic parameters in experimental animals. Fasting glucose values are presented as baseline (before the experimental protocol and dietary regimen) and after diabetes induction, while insulin and HOMA-IR were assessed at the end of the experimental period. Data are expressed as mean ± standard deviation (SD), with minimum and maximum values.

Parameter	Mean ± SD	Min-Max
Fasting glucose (baseline, mg/dL)	98.0 ± 14.1	82–119
Fasting glucose (after diabetes induction, mg/dL)	192.6 ± 86.3	125–326
Insulin (μIU/mL)	6.64 ±1.16	5.2–8.3
HOMA-IR	3.18 ± 1.38	1.8–5.6

## Data Availability

All original raw and analyzed data are deposited in the Cloud provided by WesternU and are also available with the corresponding author upon request through the proper channel.
